# Lymph Node Harvest in Dukes' A Cancer Pathologist May Need to Consider Fat Dissolving Technique: An Observational Study

**DOI:** 10.1100/2012/919464

**Published:** 2012-05-03

**Authors:** A. P. Saklani, T. Udy, T. V. Chandrasekaran, M. Davies, J. Beynon

**Affiliations:** ^1^Department of Colorectal Surgery, Abertawe Bro Morgannwg University Hospital Trust, Singleton Hospital, Swansea SA2 8QA, UK; ^2^Department of Colorectal Surgery, Abertawe Bro Morgannwg University Hospital Trust, Princess of Wales Hospital, Bridgend CF31 1RQ, UK

## Abstract

*Background*. National institute of clinical excellence (NICE) recommends that a median of 12 lymph nodes be examined in patients operated on with curative intent- to- treat colorectal cancer (CRC). Patients with lymph node harvest less than this may be considered under staged and may receive adjuvant chemotherapy. The aim of our study was to ascertain median number of lymph nodes examined in early colorectal cancers. *Method*. Patients undergoing colorectal resection between June 2007 and May 2008 were identified and pathological staging obtained using pathology database. *Results*. 146 patients underwent standardised laparoscopic or open resection of colorectal cancers during this period. Overall median number of lymph nodes harvested/patient was 14 (3–40). When analysed by stage, median number of lymph nodes harvested in Dukes' A, B, and C cancers was 10, 14, and 15, respectively. 11/18 (61%) patients with Dukes' A carcinoma had lymph node harvest of less than 12 compared with 15/55 (27%) patients with Dukes' B. *Conclusion*. Lymph node harvest in Dukes' A cancers using standard techniques tends to be low. Pathologists may have to consider special techniques in harvesting lymph nodes for early colorectal cancers.

## 1. Background

National institute of clinical excellence (NICE) Colorectal Cancer Guidance and the American Joint Committee on Cancer (AJCC) have recommended that a median of 12 lymph nodes should be examined in patients operated on with curative intent-to-treat colorectal cancer (CRC). A lymph node harvest of less than 12 will be considered below recommended standard and may lead to administration of adjuvant chemotherapy.

## 2. Aim

To assess the number of lymph nodes examined and reported in Dukes' A and Dukes' B carcinoma in regional cancer centre.

## 3. Method

Patients undergoing colorectal resection (only adenocarcinomas) with curative intent between June, 2007 and May, 2008 were identified. Squamous carcinomas of rectum and carcinoids were excluded, as adjuvant therapy after surgery in these cancers is not standardised. Like most centres in UK, majority of resections were performed open in 2007-2008, as laparoscopic colorectal surgery was being established in our unit. The pathology records of these patients was analysed retrospectively to assess the Dukes' stage and the number of lymph nodes harvested.

## 4. Results

146 patients underwent potentially curative resection for colorectal cancers (adenocarcinomas) during this period, Dukes' A 18, Dukes' B 55, and Dukes' C 73. During this period, 8.5% of all resections were laparoscopic assisted. Median number of lymph nodes harvested was 14 (3–40).

Median number of lymph nodes as per stage was Dukes' A 10.5 (3–26), B 14 (4–40), and C 15 (5–32) Table 1. Similarly median number of lymph nodes harvested and lymph node metastasis as per T stage was assessed (Table 2).

11/18 (61%) patients with Dukes' A carcinoma had lymph nodes harvest of less than 12 compared with 15/55 (27%) patients with Dukes' B. (*P* < 0.05). See Table 3.

 6 patients with rectal cancer underwent preoperative chemoradiotherapy. Even if these patients were excluded, 10/16 (62.5%) patients with Dukes' A carcinoma had lymph nodes harvest of less than 12 compared with 13/51 (25.4%) patients with Dukes' B. (*P* < 0.05). See Table 4.

## 5. Discussion

In the national bowel cancer audit project (NBOCAP), 10.5% of all colorectal cancers were diagnosed to be Dukes' A cancer [1]. National bowel cancer screening aims to detect Colorectal cancers at an earlier stage and bring about 15% reduction in mortality due to CRC. In Nottingham, population based screening, in the first round of screening 46% of detected cancers were Dukes' A [2].

At present adjuvant chemotherapy is offered to all patients with Dukes' C cancer as it reduces the recurrence rate by 41% and the death rate by 33% [3]. In patients with Dukes' B cancer, adjuvant chemotherapy offers 3.6% absolute improvement in survival, hence adjuvant chemotherapy is offered to selected patients with poor prognostic factors [4].

Staging of CRC is based on identification of lymph nodes metastasis, which in turn would depend on lymph nodes harvest and their identification by pathologists. Numerous studies have shown, patients with Dukes B cancer with lymph nodes harvest less than 8 or 9 have survival equivalent to Dukes' C cancer, suggesting under staging of cancer due to inadequate lymph nodes harvest [5–7].

In patients with colon cancer who are treated with curative intent, 12 or more nodes should normally be examined; if the median number is consistently below 12, the surgeon and the histopathologist should discuss their techniques [8]. In fact, in one recent study, lymph nodes harvest in a unit varied according to the reporting pathologist but not the operating surgeon [9].

Various societies, namely, ACPGBI, SAGES have recommended minimum of lymph nodes harvest to be 12 as quality indicator of surgery. Patients with lymph nodes harvest less than 12 may be an indicator of inadequate sampling and patients may be offered chemotherapy. While this may be appropriate for Dukes' B (T3 and T4 cancers), where incidence of lymph nodes harvest and metastasis is higher, analogy of this to T1/T2 cancers may not be appropriate where lymph nodes metastasis rate is low [10, 11]. In our study, incidence of lymph nodes metastasis in T1 (21%) and T2 (30%) cancers was higher than reported, but absolute numbers are low.

Our study shows median number of lymph nodes harvest for Dukes' A cancers was 10.5 in fact, if this was taken as inadequate sampling, 62% patients with Dukes' A cancer would have been offered chemotherapy. In reality, only one patient with Dukes' A received chemotherapy as a result of inadequate lymph nodes harvest. While the absolute number of patients with Dukes' A is about 18 (12.3%). This number is bound to increase to 46% of all cancers once screening gets underway [2].

In the 1997, TNM classification, of both the American Joint Committee on Cancer (AJCC) and the International Union Against Cancer (UICC), surgery for colorectal cancer in UK is standardised, where histological examination would ordinarily include 12 (for colorectal carcinoma) or more lymph nodes. A communication later by UICC and AJCC regarding the same cautioned against classifying less than 12 lymph nodes as Pnx. They clarified that the above statement was not intended to be a requirement for pNo but rather a guideline [12].

While the surgical resection for right hemicolectomy in UK remains standard. We can, however, improve on pathological assessment of lymph nodes. While we do agree most pathologists diligently do lymph nodes identification, special techniques to identify lymph nodes in T1/T2 cancer may be required. Special techniques like fat dissolving technique have been shown in many studies to improve lymph nodes harvest [12]. We suggest these techniques be used to improve lymph nodes identification in Dukes A cancer (T1/T2) cancers, where lymph nodes harvest was less than 12.

## 6. Conclusion

Lymph nodes harvest in Dukes' A cancer tends to be low. Pathologist may have to consider specialised techniques like fat dissolution to harvest more lymph nodes in Dukes A (T1/T2 cancers), where lymph nodes harvest is less than 12. This will assume importance as National screening programme will identify more early colorectal cancers.

## Figures and Tables

**Table 1:** Relation of number of lymph node harvested to Duke's Stage.

	Duke's A	Duke's B	Duke's C
No of patients (%)	**18 (12.3%)**	55 (37.7%)	73 (50%)
Median no lymph nodes harvested	**10.5**	14	15
Range of lymph node harvested	3–26	4–40	5–32

**Table 2:** Relation of lymph node metastasis and T stage.

	T1	T2	T3	T4
No of patients (%)	**14 (9.6%)**	**10 (6.8%)**	91 (62.3%)	31 (21.2%)
Median no lymph nodes harvested	**11.5**	**11.0**	15	17
Metastatic lymph node (%)	3/14 (21%)	3/10 (30%)	44/91 (48%)	23/31 (74%)

**Table 3:** Corelation between lymph node harvest and Dukes' stage.

All patients	Lymph nodes harvest < 12	Lymph nodes harvest ≥ 12	Total
Dukes' A	11	7	18
Dukes' B	15	40	55

Total	26	47	73

**Table 4:** Corelation between lymph node harvest and Dukes' stage (6 patients undergoing radiotherapy excluded).

Patients undergoing Radiotherapy excluded	Lymph nodes harvest < 12	Lymph nodes harvest ≥ 12	Total
Dukes' A	10	6	16
Dukes' B	13	38	51

Total	23	44	67
